# Stimulation of α7-nAChRs coordinates autophagy and apoptosis signaling in experimental knee osteoarthritis

**DOI:** 10.1038/s41419-021-03726-4

**Published:** 2021-05-05

**Authors:** Yuan Liu, Shi Xu, Haijun Zhang, Kaoliang Qian, Jiachen Huang, Xianger Gu, Yan Li, Yi Fan, Jun Hu

**Affiliations:** 1 Department of Infectious Diseases, The First Affiliated Hospital of Nanjing Medical University, 210029 Nanjing, China; 2 Department of Pharmacology, Nanjing Medical University, 211166 Nanjing, China; 3 Department of Orthopedics, The First Affiliated Hospital of Nanjing Medical University, 210029 Nanjing, China

**Keywords:** Autophagy, Apoptosis

## Abstract

Osteoarthritis (OA) is the most common chronic joint disease in the elderly population. Growing evidence indicates that a balance between autophagy and apoptosis in chondrocytes plays a key role in OA’s cartilage degradation. Thus, drugs targeting the balance between apoptosis and autophagy are potential therapeutic approaches for OA treatment. In previous studies, we found that the activation of α7 nicotinic acetylcholine receptors (α7-nAChRs) alleviated monosodium iodoacetate (MIA)-induced joint degradation and osteoarthritis pain. To explore the potential functions of α7-nAChRs in autophagy and apoptosis signaling in knee OA, we compared the expression of α7-nAChRs in human knee articular cartilage tissues from normal humans and OA patients. We found that knee joint cartilage tissues of OA patients showed decreased α7-nAChRs and an imbalance between autophagy and apoptosis. Next, we observed that α7-nAChRs deficiency did not affect cartilage degradation in OA development but reversed the beneficial effects of nicotine on mechanical allodynia, cartilage degradation, and an MIA-induced switch from autophagy to apoptosis. Unlike in vivo studies, we found that primary chondrocytes from α7-nAChRs knockout (KO) mice showed decreased LC3 levels under normal conditions and were more sensitive toward MIA-induced apoptosis. Finally, we found that α7-nAChRs deficiency increased the phosphorylation of mTOR after MIA treatment, which can also be observed in OA patients’ tissues. Thus, our findings not only confirmed that nicotine alleviated MIA-induced pain behavior and cartilage degradation via stimulating the α7-nAChRs/mTOR signal pathway but found the potential role of α7-nAChRs in mediating the balance between apoptosis and autophagy.

## Introduction

Osteoarthritis (OA), a chronic degenerative joint disease, is mainly characterized by cartilage degradation, subchondral bone sclerosis, synovial inflammation, and osteophyte formation. It most commonly affects weight-bearing and high-use joints, including those of the hip, knees, and hands^1^. OA is a leading cause of disability among older adults, affecting their physical health and mental health^2^. With the aging of modern society, OA is likely to cause increased morbidity and higher healthcare costs worldwide^3^.

A balance between autophagy and apoptosis in chondrocytes plays a key role in the cartilage degradation of OA. Apoptosis is a physiological process of programmed cell death. It is regulated by many factors and is highly involved in the development and growth of the human body, homeostasis of the internal environment, and the aging process. Abnormal apoptotic processes can lead to various pathological conditions, including tumors, dysplasia, and various degenerative diseases^4^. Different studies have reported that the rate of apoptotic chondrocytes in OA cartilage ranges from <1% to ~20%^5,6^. Numerous empty lacunae and decreased chondrocyte numbers in the calcified cartilage layer were observed compared to those in other cartilage layers, indicating the relative contribution of chondrocyte apoptosis in pathogenesis OA^7^. Recently, autophagy, a cellular self-digestion process, has been intimately connected with chondrocyte apoptosis in OA^8^. In the aging process^9^ and OA mouse models^10^, the expression of autophagy proteins ULK1 (unc-51 like autophagy activating kinase 1) and Beclin-1 was decreased, accompanied by increased apoptosis. Furthermore, rapamycin-induced autophagy ameliorated cartilage degradation in OA-like lesions^11^ and mouse models^12^. These results suggest that drugs targeting the balance between apoptosis and autophagy may be potential therapeutic approaches for OA treatment.

A meta-analysis revealed an inverse association between cigarette smoking and the risk of knee OA in males, irrespective of study design^13^. A series of retrospective analyses of the association between smoking and OA points toward an important etiologic clue, indicating that some particular bioactive substances in tobacco help alleviate cartilage degradation associated with OA^13–15^. Nicotine, a key component in tobacco products, may serve as a potential therapeutic agent for OA via activating alpha-7 nicotinic acetylcholine receptors (α7-nAChRs)^16–18^. α7-nAChRs are recognized to have essential immunological anti-inflammatory roles in the human body in recent years^19–22^. We also found that α7-nAChRs activation with nicotine alleviated monosodium iodoacetate (MIA)-induced joint degradation^23^ and OA pain^18^. However, little is known about the role of α7-nAChRs in autophagy and apoptosis signaling in knee OA.

In the present study, using our previously established in vivo and in vitro models and an α7-nAChR knockout (KO) mouse model, we investigated whether autophagy and apoptosis were involved in the progression of MIA-induced OA lesions and further observed whether α7-nAChRs activation participated in coordinating autophagy and apoptosis signaling in OA progression.

## Results

### Articular cartilage tissues of OA patients showed decreased α7-nAChRs and an imbalance between autophagy and apoptosis

**Fig. 1:**
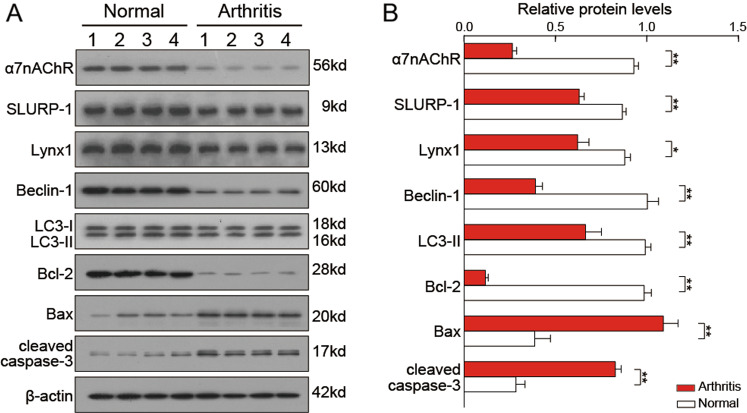
Expression of α7-nAChRs and some autophagy and apoptosis markers in human normal and OA cartilage tissues. Immunoblots of lysates obtained from human normal and OA cartilage probed with an antibody for α7-nAChRs, SLURP-1, Lynx1, Beclin-1, LC3-I/II, Bcl-2, Bax, or cleaved caspase-3, and β-actin as a loading control (**A**). In cartilage tissues of OA patients, the levels of pro-apoptotic protein Bax and cleaved caspase-3 were significantly increased, while those of anti-apoptotic protein Bcl-2 and autophagy markers Beclin-1 and LC3-II were significantly reduced (**B**). The results shown represent mean ± SEM from four samples. In (**B**), **P < 0.01, *P < 0.05 versus normal tissues, using Student’s unpaired *t* test.

To evaluate the roles of α7-nAChRs in OA development, we compared the expression of α7-nAChRs and some autophagy and apoptosis markers in human articular cartilage tissues between normal tissues and tissues from OA patients. As showed in Fig. 1, the levels of α7-nAChRs and two endogenous cholinergic modulators SLURP-1 (Secreted mammalian Ly6/urokinase plasminogen activator receptor-related protein-1)^24^ and Lynx1 (Ly6/Neurotoxin 1)^25^ was significantly decreased (*P* < 0.05) in OA patients, indicating the dysfunction of the nicotinic cholinergic system. As expected, the levels of pro-apoptotic protein Bax and cleaved caspase-3 were significantly increased (*P* < 0.01) in OA patients, while that of anti-apoptotic protein Bcl-2 was significantly reduced (*P* < 0.01) (Fig. 1A, B). Also, the levels of autophagy markers Beclin-1 and LC3-II were significantly decreased (61.0% and 32.9%, respectively) in the tissues of OA patients (Fig. 1A and B). These results indicated a switch from autophagy to apoptosis during OA progression.

### Genetic deficiency of α7-nAChRs did not affect the progress of OA but diminished nicotine-attenuated mechanical allodynia and cartilage degradation

**Fig. 2:**
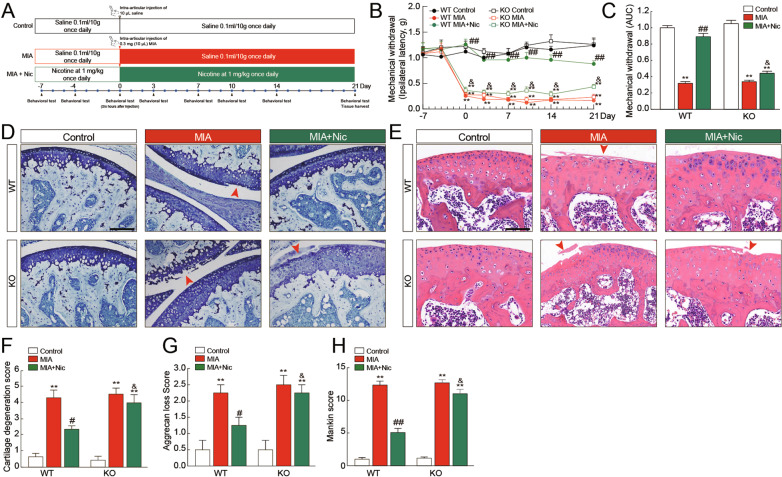
Effects of deficiency of α7-nAChRs on mechanical allodynia and cartilage degradation in OA mice. After receiving intraperitoneal *(i.p)* injections of 1 mg/kg nicotine or saline once daily for 7 days, both wild-type (WT) mice and α7-nAChRs knockout (KO) mice were injected with 0.3 mg/10 μL MIA in the right knee joint and were injected with nicotine or saline once daily for 3 weeks (**A**). Mechanical allodynia was measured as a reduced threshold to von Frey hairs (**B**). The results are represented as mean ± SEM in 10 mice per group. **P < 0.01 versus corresponding control group; ^##^*P* < 0.01 versus corresponding MIA group; ^&^*P* < 0.05 versus WT MIA^+^Nic group, using a two-way repeated-measures ANOVA followed by Tukey’s test. The deficiency of α7-nAChRs did not affect an MIA-induced reduction in withdrawal threshold but diminished the effect of nicotine on MIA-induced mechanical allodynia (**C**). The results are represented as mean ± SEM in 10 mice per group. **P < 0.01 versus corresponding control group; ^##^*P* < 0.01 versus corresponding MIA group; ^&^*P* < 0.05 versus WT MIA + Nic group, using a two-way ANOVA followed by Tukey’s test. There were representative sections of toluidine blue staining (**D**) and hematoxylin-eosin staining (**E**) from WT mice and KO mice. Scale bar = 100 μm. The red arrow indicates that α7-nAChR deficiency in KO mice showed no difference in toluidine blue staining and hematoxylin-eosin staining than WT mice after MIA injection. Nicotine could reduce the MIA-induced increase of cartilage degeneration score (**F**), aggrecan loss score (**G**), and Mankin score (**H**) in WT mice but not in α7-nAChR KO mice. The results are represented as mean ± SEM in 4 mice per group. **P < 0.01 versus corresponding control group; ^##^*P* < 0.01, ^#^*P* < 0.05 versus corresponding MIA group; ^&^*P* < 0.05 versus WT MIA + Nic group, using a two^-^way ANOVA followed by Tukey’s test.

To evaluate the functional role of α7-nAChRs in OA in vivo, we studied the development of MIA‐induced OA‐related changes in α7-nAChRs KO mice and their WT littermates (Fig. 2A). MIA, injected for 3 weeks in mice, induced robust mechanical allodynia, measured as a reduced threshold to von Frey hairs (Fig. 2B and C). However, there was no difference in the threshold between KO mice and their WT littermates following MIA injection. To further ascertain the effects of α7-nAChRs on cartilage degradation, cartilage degeneration score, aggrecan loss score, and Mankin score of knee joints were estimated. No difference in toluidine blue staining (Fig. 2D) and hematoxylin-eosin staining (Fig. 2E) was observed between KO mice and age-matched WT mice after MIA injection (*P* > 0.05). Consistent with the finding in immunohistochemistry, there was no difference in the cartilage degeneration score (Fig. 2F), aggrecan loss score (Fig. 2G), and Mankin score (Fig. 2H). As expected, nicotine dramatically attenuated MIA-induced mechanical allodynia and cartilage degeneration in WT mice, but this attenuation trend was not observed in KO mice. These results indicate that deletion of α7-nAChRs did not affect cartilage degradation in OA development, although it diminished the effects of nicotine in the MIA-induced OA mouse model.

### Genetic deficiency of α7-nAChRs diminished the modulation of apoptosis and autophagy in OA mice caused by nicotine

**Fig. 3:**
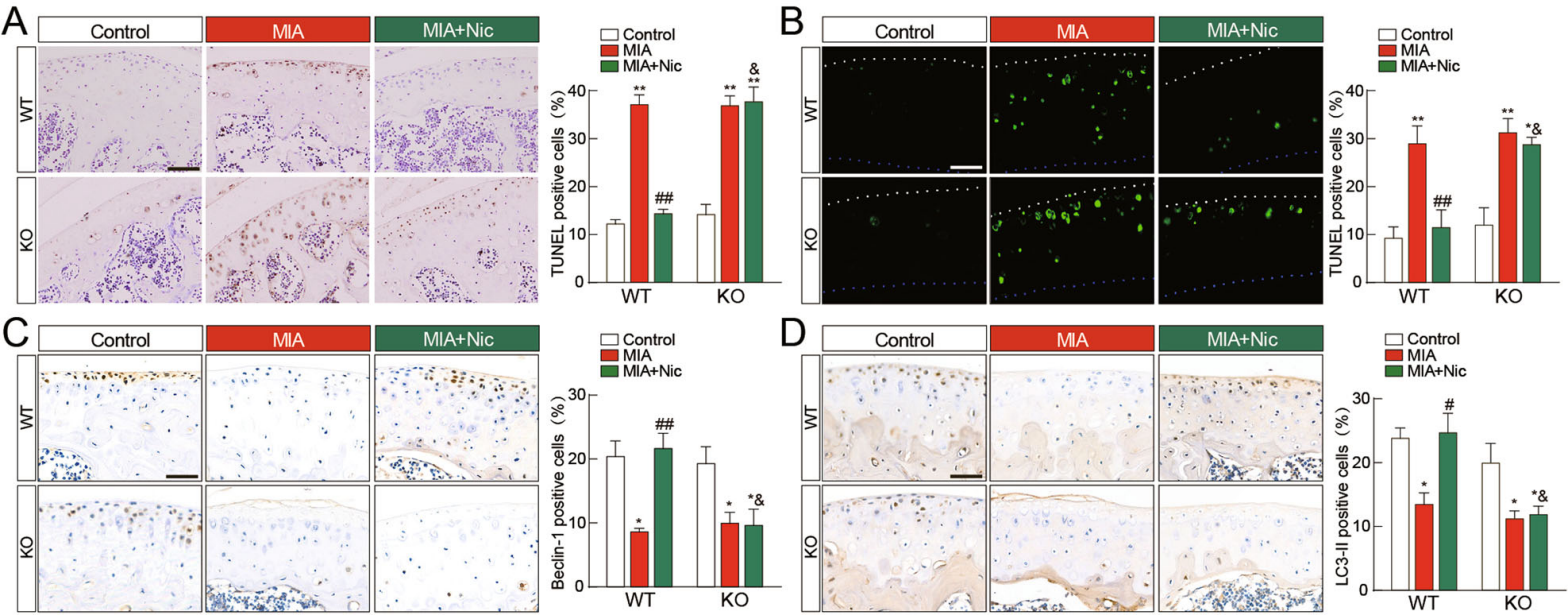
Effects of α7-nAChRs deficiency on chondrocyte apoptosis and autophagy in OA mice. Representative sections of TUNEL^+^ (**A** for HRP-DAB detection and **B** for fluorescence detection), Beclin-1^+^ (**C**), or LC3 ^+^ (**D**) staining from wild-type (WT) mice and α7-nAChR knockout (KO) mice. Scale bar = 100 μm. MIA injection increased the number of TUNEL^+^ cells (**A** and **B**) and reduced the numbers of Beclin-1^+^ (**C**) and LC3 ^+^ cells (**D**) with no significant differences between WT and KO mice. α7-nAChR deletion diminished the effects of nicotine on the modulation of apoptosis and autophagy after MIA injection. The results are represented as mean ± SEM in 4 mice per group. **P < 0.01, *P < 0.05 versus corresponding control group; ^##^*P* < 0.01 versus corresponding MIA group; ^&^*P* < 0.05 versus WT MIA + Nic group^,^ using two-way ANOVA followed by Tukey’s test.

To determine the effects of α7-nAChRs on MIA-induced chondrocyte apoptosis and autophagy in vivo, we counted the numbers of TUNEL^+^, Beclin-1^+^, and LC3^+^ cells in the knee joints. There was no difference in the numbers of TUNEL^+^ (Fig. 3A for HRP-DAB detection and Fig. 3B for fluorescence detection), Beclin-1^+^ (Fig. 3C), and LC3^+^ (Fig. 3D) cells in both the genotypes of control mice. Consistent with cartilage degradation, no significant differences in the MIA-induced increase of TUNEL^+^ cells and the reduction of Beclin-1^+^ and LC3^+^ cells were observed between KO and WT mice. As expected, nicotine treatment significantly reduced TUNEL^+^ cells by 61.4% and 59.8% (HRP-DAB and fluorescence staining, respectively) than by treatment with MIA alone. Moreover, nicotine treatment increased Beclin-1^+^ cells by 247.5% and LC3^+^ cells by 191.5% than with MIA alone in WT mice but not in KO mice. Thus, these findings suggest that α7-nAChRs may not be directly involved in MIA-induced OA development but mediate the effects of nicotine on the switch from autophagy to apoptosis.

### Chondrocytes from α7-nAChR KO mice showed increased susceptibility to MIA-induced apoptosis and a reduction in LC3 levels

**Fig. 4:**
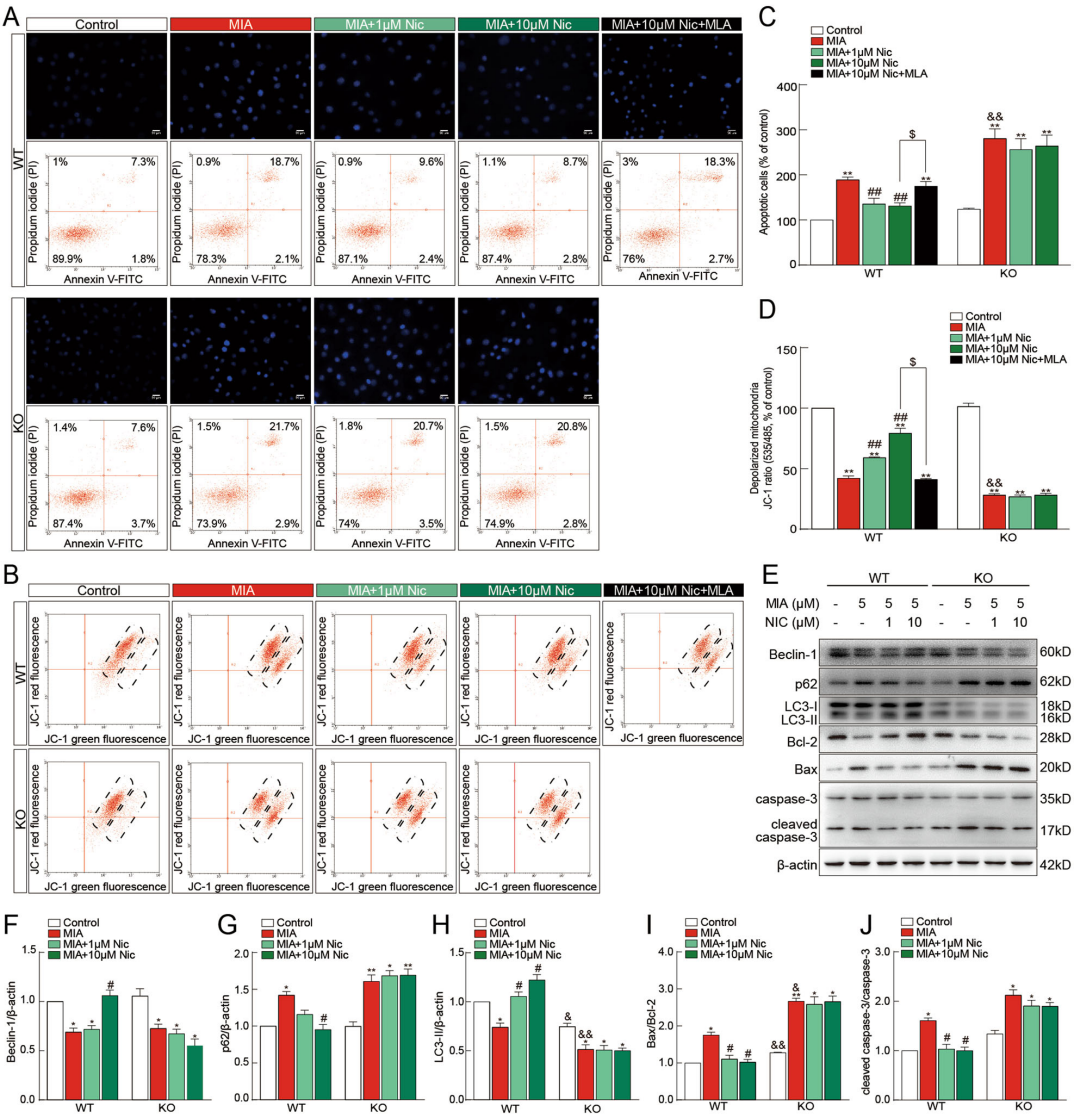
Effects of α7-nAChRs deficiency on apoptosis and autophagy in primary cultured chondrocytes. Representative images of Hoechst 33342 staining (upper, **A**) and flow cytometry (lower, **A**) from wild-type (WT) mice and α7-nAChR knockout (KO) mice. Scale bar = 50 μm. The loss of mitochondrial membrane potential (ΔΨm) in chondrocytes receiving the indicated treatments is depicted using the JC-1 dye (**B**). The apoptotic index (**C**) and the measurement of ΔΨm (**D**) determined by flow cytometry showed that KO chondrocytes were more susceptible to MIA-induced apoptosis. Immunoblots of lysates obtained from cultured chondrocytes probed with antibodies to Beclin-1, p62, LC3 (I and II), Bcl-2, Bax, cleaved caspase-3, caspase-3, or β-actin as a loading control are shown (**E**). The levels of an autophagy substrate p62 (**G**), the Bax/Bcl-2 ratio (**I**), and cleaved caspase-3/caspase-3 ratio (**J**) were significantly increased in KO chondrocytes after MIA treatment, while LC3-II levels of KO chondrocytes were significantly lower than those of WT chondrocytes (**H**). Both α7-nAChR deficiency (**A**, **B**, **C**, **D**, **E**, **F**, **G**, **H**, **I**, and **J**) and α7-nAChR antagonist MLA (**A**, **B**, **C**, and **D**) could diminish the protective effect of nicotine on chondrocyte apoptosis. The results are represented as mean ± SEM of three independent experiments in (**C**, **D**, and **F–J**). **P < 0.01, *P < 0.05 versus corresponding control group; ^##^ *P* < 0.01, ^#^*P* < 0.05 versus corresponding MIA group; ^&&^*P* < 0.01, ^&^*P* < 0.05 versus WT MIA group, ^$^*P* < 0.05 versus WT MIA + 10 μM nicotine, using two-way ANOVA followed by Tukey’s test.

To further confirm the involvement of α7-nAChRs in chondrocyte degradation, we assessed whether there was an MIA-induced chondrocyte difference in vitro. Unlike in vivo studies, KO chondrocytes were more sensitive toward MIA-induced apoptosis and showed an increased apoptosis rate (*P* < 0.01; Fig. 4A and C) and a reduced mitochondrial membrane potential (*P* < 0.05; ΔΨm; Fig. 4B and D) after MIA stimulation. Moreover, compared to WT chondrocytes, KO chondrocytes treated with MIA showed higher expression of p62 (*P* < 0.05; Fig. 4G), increased Bax/Bcl-2 ratio (*P* < 0.01; Fig. 4I), and enhanced cleaved caspase-3/caspase-3 ratio (*P* < 0.05; Fig. 4J), while reduced expression of LC3. Interestingly, under normal conditions, the expression of both LC3-I and LC3-II in KO chondrocytes was lower compared to cells from WT controls (*P* < 0.05) (Fig. 4H). As expected, nicotine treatment could statistically decrease cell apoptosis, alleviate ΔΨm loss, stabilize the Bax/Bcl-2 balance, and inhibit cleaved caspase-3 activity via α7-nAChRs, while promoting autophagy via inhibiting p62 activity and increasing the levels of Beclin-1 and LC3-II. Both α7-nAChRs deficiency and the α7-nAChR antagonist MLA diminished the protective effect of nicotine in vitro. These findings indicated a different role of α7-nAChRs on chondrocyte degradation in vitro, especially on the LC3 levels.

### α7-nAChRs activation promoted autophagy by regulating the phosphorylation of mTOR

**Fig. 5:**
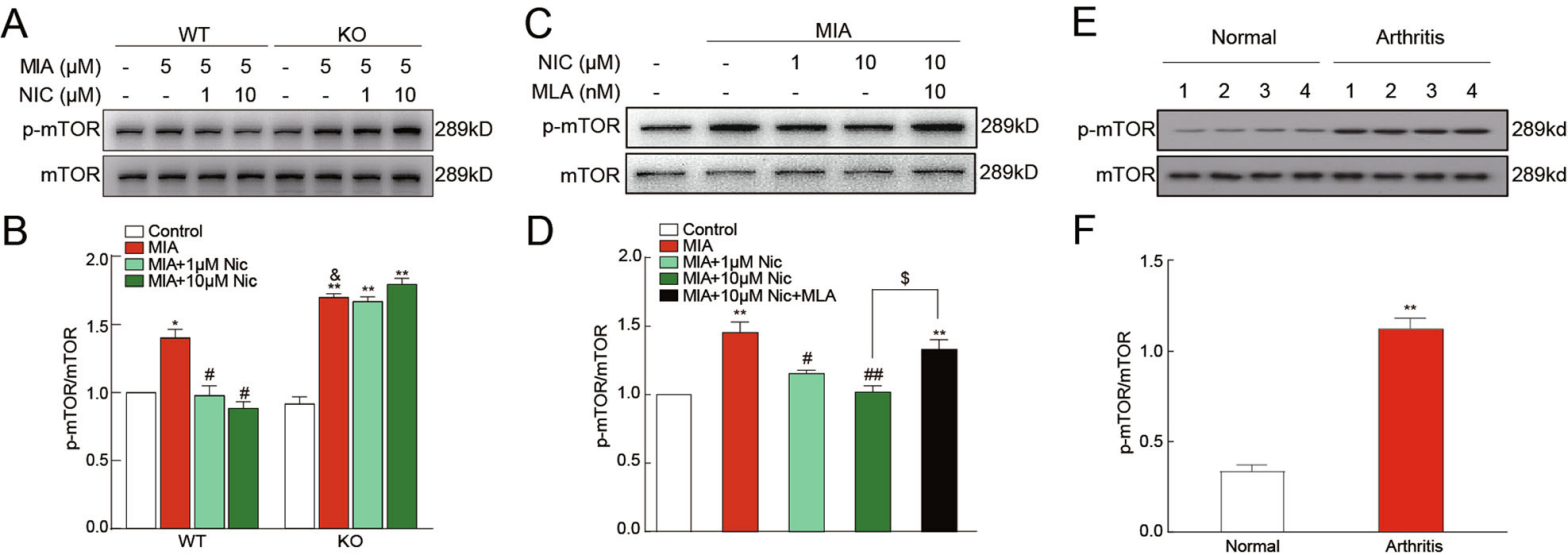
Effects of α7-nAChRs activation on phosphorylation of mTOR in primary cultured chondrocytes and human cartilage tissues. Immunoblots of lysates obtained from cultured wild-type (WT) chondrocytes and α7-nAChR knockout (KO) chondrocytes probed with antibodies to p-mTOR or mTOR (**A**). Compared to that in WT chondrocytes, the phosphorylation of mTOR was increased in KO chondrocytes after MIA treatment (**B**). Immunoblots of lysates were obtained from cultured WT chondrocytes treated with nicotine (Nic) or Nic + MLA probed with antibodies to p-mTOR or mTOR (**C**). Nic reduced the intensity of MIA-induced phosphorylation of mTOR (**B** and **D**), which could be reversed by MLA (**D**). Immunoblots of lysates obtained from human cartilage tissues probed with antibodies to p-mTOR or mTOR (**E**). Phosphorylation of mTOR in tissues of OA patients was significantly increased (**F**). The results are represented as mean ± SEM of three independent experiments in (**B** and **D**), 4 samples/group in (**F**). **P < 0.01, *P < 0.05 versus corresponding control group; ^##^*P* < 0.01, ^#^*P* < 0.05 versus corresponding MIA group; ^&^*P* < 0.05 versus WT MIA group; ^$^*P* < 0.05 versus WT MIA + 10 μM nicotine, using two-way ANOVA followed by Tukey’s test (**B**), one-way ANOVA followed by Tukey’s test (**D**), or Student’s unpaired *t* test (**F**).

**Fig. 6:**
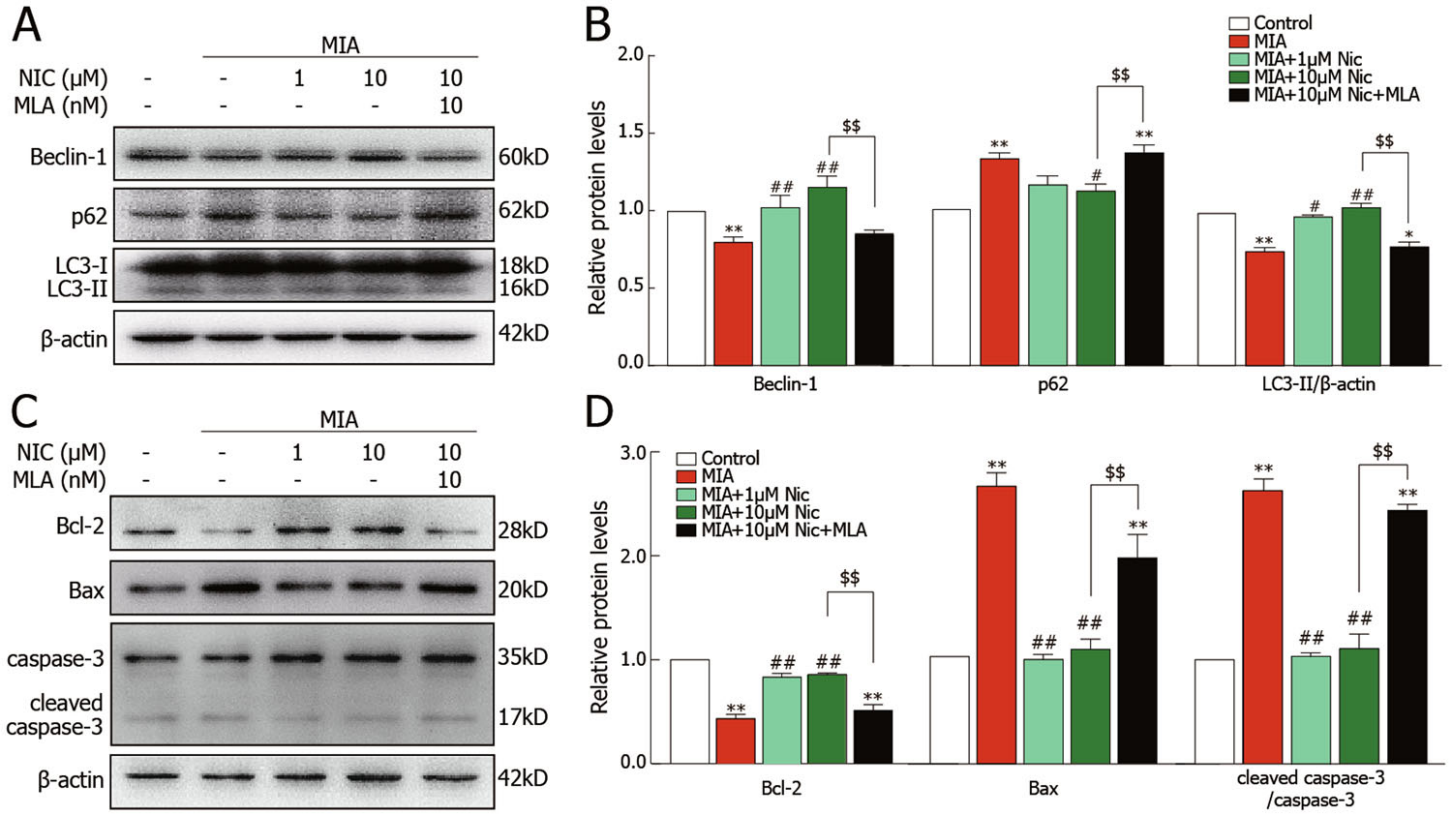
Effects of nicotine on autophagy- and apoptosis-related proteins in primary cultured wild-type chondrocytes. (**A**) Immunoblots of lysates obtained from cultured chondrocytes probed with antibodies to Beclin-1, p62, LC3, or β-actin as a loading control. (**B**) The MIA-induced reduction of Beclin-1 and LC3 and the increase of p62 levels were significantly decreased by 10 μM nicotine, which MLA could reverse. (**C**) Immunoblots of lysates obtained from cultured chondrocytes probed with antibodies against Bcl-2, Bax, cleaved caspase-3, and caspase-3. (**D**) The MIA-induced increase in Bax and the cleaved caspase-3 / caspase-3 ratio and the reduction of Bcl-2 were significantly decreased by 10 μM nicotine, which MLA could reverse. **P < 0.01, *P < 0.05 versus control group; ^##^*P* < 0.01, ^#^*P* < 0.05 versus MIA group; ^$$^*P* < 0.01 versus MIA + 10 μM nicotine by one-way ANOVA followed by Tukey’s test.

mTOR is one of the most important autophagy regulators in cells. Studies have shown that mTOR might play an important role in modulating autophagy and apoptosis in chondrocytes^26^. We next examined whether the mTOR signaling pathway contributes to the protective effects of α7-nAChRs against MIA-induced switch from autophagy to apoptosis in chondrocytes. Compared to WT chondrocytes, the phosphorylation of mTOR was increased in KO chondrocytes after MIA treatment (Fig. 5A and B). Nicotine (10 μM) could inhibit the p-mTOR by 22.2% in WT chondrocytes, while it could not modulate p-mTOR in KO chondrocytes. Moreover, MLA (10 nM) abolished the effects of nicotine on MIA-induced phosphorylation of mTOR (Fig. 5C and D). We also found that the levels of p-mTOR in tissues of OA patients were significantly increased by 333.5% (Fig. 5E and F). Meanwhile, MLA (10 nM) abolished the effects of nicotine on the autophagy pathway (Beclin-1, p62, and LC3-II; Fig. 6A and B) and the apoptosis pathway (Bax, Bcl-2, and cleaved caspase-3; Fig. 6C and D). These findings collectively implied that the mTOR pathway might be a key link in the association between autophagy and apoptosis via α7-nAChR activation.

## Discussion

The cardinal feature of cartilage degeneration in OA is the death of chondrocytes, which are essential for regulating matrix synthesis and degradation. Different types of cell death in cartilage have been reported, including apoptosis and autophagy, which might play pivotal roles in the progression of OA. In the present study, we confirmed that a switch from autophagy to apoptosis occurred in articular cartilage during the progression of OA and discovered for the first time a reduction in the expression of α7-nAChRs and its endogenous allosteric modulators in human articular cartilage tissues from OA patients. Secondly, we revealed that nicotine protects against the adverse effects of MIA in OA mouse models by modulating autophagy and apoptosis in OA chondrocytes through the activation of α7-nAChRs. However, we found that α7-nAChRs deficiency did not affect MIA-induced OA development in vivo but mediate the switch from autophagy to apoptosis in primary chondrocytes. Unlike in vivo studies, we found a different role of α7-nAChRs on chondrocyte degradation in vitro, especially on the LC3-II levels. Finally, we found that the mTOR pathway might be a key link in the association between autophagy and apoptosis via α7-nAChR activation. These results collectively suggest that activation of α7-nAChR signaling might play a pivotal role in coordinating autophagy and apoptosis signaling in the progression of MIA-induced OA.

The aging of joints is characterized by the loss of progressive functional and physiological integrity caused by multiple factors, including genomic and epigenetic changes and cellular senescence^27^. Hashimoto et al. revealed that 22.3% of OA chondrocytes undergo apoptosis than 4.8% of normal chondrocytes^28^. The number of apoptotic cells was significantly correlated with the grade of OA. Therefore, chondrocyte apoptosis might be a valid target to modulate cartilage degeneration in human OA. Recently, the pro-survival functions of α7-nAChRs were proved by more and more articles^29,30^. Yuan et al. found that nicotine suppresses H_2_O_2_-induced mouse brain astrocyte apoptosis through the stimulation of α7-nAChRs^29^. Shi et al. found that a selective α7-nAChR agonist PNU-282987 could afford neuroprotection and result in a reduction in apoptotic SH-SY5Y cell death^30^. However, little is known about the role of α7-nAChRs in autophagy and apoptosis signaling in knee OA. In the present study, we showed that nicotine inhibited MIA-induced apoptosis of articular chondrocytes, and this anti-apoptotic effect of nicotine could be abolished by KO of α7-nAChRs or by treatment with MLA, implying that α7-nAChRs play a critical role in chondrocyte apoptosis. We also found that stabilization of mitochondrial membrane potential of chondrocytes, balance in Bax/Bcl-2 ratio, and caspase-3 cleavage inhibition were involved in anti-apoptotic mechanisms of nicotine via α7-nAChRs, suggesting that mitochondrial apoptotic pathways might be involved in the anti-apoptotic effect of nicotine via α7-nAChRs. Meanwhile, α7-nAChRs are expressed in both the plasma membrane and mitochondria outer membrane^31^ and are involved in regulating mitochondrial permeability by directly affecting the pore-forming Bax and voltage-dependent anion channels (VDAC1)^32^. Moreover, α7-nAChR expression was shown to be related to Bax expression^33^. Thus, α7-nAChRs in mitochondria outer membrane plays a critical role in the anti-apoptotic mechanisms of nicotine, but whether the plasma membrane α7-nAChRs also involved in the anti-apoptotic effects need to be further studied.

A growing number of studies have shown that, in OA, the reduction of critical autophagy regulators is accompanied by increased cell apoptosis, which can aggravate cartilage degradation. As a conserved protein degradation pathway in eukaryotic cells, autophagy can save and provide energy for cells by degrading proteins and residual organelles and can promote the degradation of apoptosis-related proteins, thereby reducing the cytotoxicity caused by these aggregated proteins^9,10,34^. For instance, p62 is a key factor in the selective autophagic degradation of many proteins and mitochondria. It can directly interact with some proteins and regulate the apoptosis signaling pathway^35^. Some studies have confirmed that the Bcl-2/Beclin-l complex, which determines whether a cell enters an apoptosis program or initiates an autophagy program, functions as a “trigger” in the occurrence and development of apoptosis and autophagy. Beclin-l can be linked to Bcl-2 by its BH3 structural domain. When Beclin-1 is released from the Bcl-2 binding protein, autophagy is activated. Death-associated protein kinase phosphorylates Beclin-1 and JNK phosphorylates Bcl-2^36^. In the present study, α7-nAChR deficiency reduced the activation of autophagy signaling pathway by nicotine, as evidenced by the reversal of nicotine-induced regulation of Beclin-1, p62, and LC3-II in mouse primary chondrocytes. Interestingly, although no difference in the numbers of Beclin-1^+^ and LC3^+^ cells in vivo, LC3-II levels in KO chondrocytes were significantly lower than that in WT chondrocytes in vitro. Chondrocyte difference between in vivo and in vitro was also found in apoptosis^37^ and cartilage growth^38^. Recently, α7-nAChRs deficient mice showed an impairment of the autophagic function via reducing the AMPK-mTOR-p70S6K signaling pathway^39^. Since we observed decreased levels of LC3 in KO chondrocytes, it is important to study further the role of α7-nAChRs in autophagy signaling in knee OA.

**Fig. 7:**
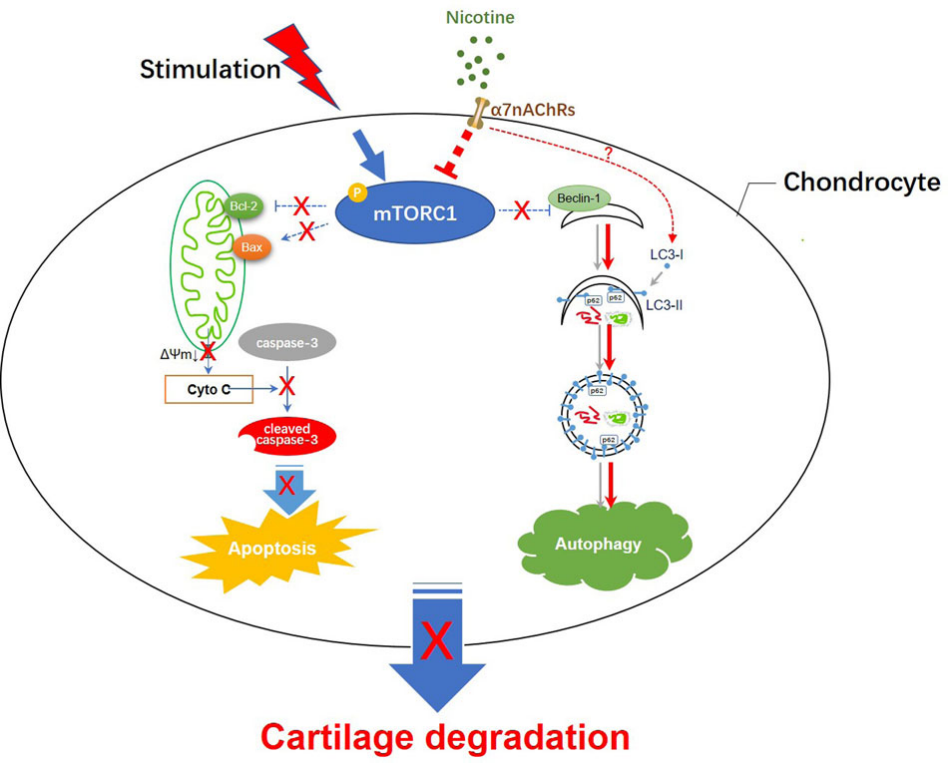
A working model shows that α7-nAChRs play a role in switching from autophagy to apoptosis in cartilage degradation of osteoarthritis (OA). In OA, some factors such as inflammatory cytokines can cause phosphorylation of mTOR in chondrocytes and switch autophagy to Bax/Bcl2/caspase-3‒mediated mitochondrial apoptosis, resulting in cartilage degradation. Activation of α7-nAChRs could inhibit the phosphorylation of mTOR, promote autophagic flux, and prevent cartilage degradation.

Some studies have demonstrated that mTOR is one of the most important autophagy regulators in cells. Activation of mTOR can inhibit autophagy induction by inhibiting Atg1-Atg13 binding. Other studies have proved that the upregulation of mTOR is related to the down-regulation of OA chondrocyte apoptosis. Compared with normal mice, mice with KO of specific gene-mTOR have higher cartilage autophagy levels and decreased chondrocyte apoptosis^40^. We also found that phosphorylation of mTOR in tissues of OA patients was significantly increased compared with that in normal cartilage tissues. Furthermore, p-mTOR was increased in KO chondrocytes than in WT mouse chondrocytes after MIA treatment. Knockout (KO) of α7-nAChRs also blocked the recovery of MIA-induced phosphorylation of mTOR by nicotine treatment in vivo and in vitro. Yang et al. found that in in vivo and in vitro studies, mTOR complex 1, downstream to p-ERN1, could not only suppress autophagy but also promote p-EIF2AK3‒mediated endoplasmic reticulum apoptosis^26^. Their results established that aberrant mechanical loading causes cartilage degeneration by activating, at least in part, the mTOR complex 1 signaling, which modulates autophagy and apoptosis in chondrocytes. Several experiments have demonstrated that inhibiting mTOR through pharmacological or genetic methods to enhance autophagy can prolong the lifespan of chondrocytes. Our working model shows that α7-nAChRs switch from autophagy to apoptosis in OA cartilage degradation (Fig. 7). Further research is needed to elucidate the relationship between α7-nAChRs and mTOR/LC3.

The limitations of this study must be acknowledged. The identified knee joint cartilage tissues of OA patients exhibited satisfactory sensitivity, specificity, and accuracy in distinguishing these patients from control patients with traumatic amputation. However, the small sample size of four OA patients versus four controls could affect the study results’ reliability because it leads to a higher variability. Therefore, in the future, we will further explore our findings by conducting studies on α7-nAChRs expression in a larger sample size. Second, mTOR links with other proteins and serves as a core component of two distinct protein complexes, mTOR complex 1 and mTOR complex 2, regulating different cellular processes^41^. Based on the previous study^26^, we focus on the mTORC1 in the current paper because mTORC1 might play an important role in regulating a switch from autophagy to apoptosis. However, mTORC2 might also involve supporting cell survival and inhibiting apoptosis, which possesses the PI3K-Akt pathway regulated by α7-nAChRs^42^. Thus, the role of mTORC1 or mTORC2 in cell death regulation mechanisms of α7-nAChRs needs to be evaluated furtherly.

In conclusion, to the best of our knowledge, we have provided the first evidence of the roles of α7-nAChRs in coordinating autophagy and apoptosis signaling in MIA-induced experimental osteoarthritic knees. Our results shed light on the therapeutic strategy of protecting articular chondrocytes in OA cartilage. It is worth noting that nicotine can interact with multiple nicotinic receptor subtypes, which may lead to uncertainty related to the role of α7-nAChRs in OA. Moreover, nicotine could stimulate prooncogenic processes^43^, so nicotine-based drugs for OA treatment should be used with caution. To resolve this contradiction, the discovery of α7-nAChRs-specific agonists without side effects will provide a direction for future research.

## Materials and methods

### Preparation of cartilage specimens

Four human cartilage specimens were retrieved from patients who underwent total knee replacement surgery, while four control specimens were obtained from individuals who underwent traumatic amputation and having neither OA nor rheumatoid arthritis. All the specimens were obtained from the Department of Orthopedics, the First Affiliated Hospital of Nanjing Medical University. Informed consent was obtained from each participant, and the procedures were approved by the Ethics Committee of the First Affiliated Hospital of Nanjing Medical University. Proteins were extracted by treating the samples with 1 mL 4 M guanidinium chloride containing 50 mM sodium acetate, 10 mM EDTA, and 0.1 M hexanoic acid pH 5.8 at 4 °C for 48 h. Each extract was separated from the cartilage residue by centrifugation (800 × *g*) at 4 °C for 10 min and stored at −70 °C before use.

### Mice

All animal experiments were approved by the Institutional Animal Care and Use Committee of Nanjing Medical University (IACUC). α7-nAChR KO mice (male, 10–12 weeks old, weighing 24–28 g, C57BL/6 J background) were purchased from the Jackson Laboratory (B6.129S7-charna7tm1bay, number 003232; Bar Harbor, ME, USA). Wild-type (WT) C57BL/6 J mice (male, 10–12 weeks old, weighing 24–28 g) were purchased from the Animal Core Facility of Nanjing Medical University (Nanjing, Jiangsu, China). Mice were maintained in an animal facility under a standardized light-dark cycle and had free access to food and water. Both genotypes of mice were randomly divided into control, MIA, and MIA + Nic groups. Behavioral testing was performed during the light cycle.

### Induction of OA and drug treatment

OA was induced by a single intra‐articular injection of (MIA, Sigma-Aldrich, St. Louis, MO, USA), as previously described^44^. Briefly, under anesthesia using isoflurane inhalation, 0.3 mg MIA in 10 μL physiological sterile saline was administered in the right knee joint of each mouse. Sham operation was performed using a similar surgery but with a 10 μL saline injection. Seven days before the MIA injection, mice in a nicotine-treated group received intraperitoneal (i.p) injections of a well-known α7-nAChRs agonist nicotine (0.5 or 1 mg/kg; Sigma-Aldrich, St. Louis, MO, USA) once daily. After the MIA injection, the mice were administered nicotine injections once daily for 3 weeks, while matched mice received an equal volume of saline. Nicotine was injected 30 min before each MIA injection. Methyllycaconitine (MLA, 1.0 mg/kg), used for nicotine antagonism studies, was injected 30 min before nicotine administration^18^.

### Behavioral analysis

Mechanical sensitivity was measured using the von Frey hair test (Woodland Hills, Los Angeles, CA, USA). Calibrated von Frey hairs (0.008 g, 0.02 g, 0.04 g, 0.07 g, 0.16 g, 0.4 g, 0.6 g, 1.0 g, 1.4 g, and 2 g fibers) were applied to the plantar surface of hind paw of mice until the fibers bent^18^. Mice were habituated to the test environment for at least 3 days before baseline testing. Next, they were placed in boxes set on an elevated metal mesh floor and allowed to habituate for 30 min before testing. Each hind paw’s plantar surface was stimulated with a series of von Frey hairs with log-arithmetical increments in stiffness, applied perpendicularly to the plantar surface. Each mouse was tested three times, and the average threshold was measured. Mice were considered allodynic when they displayed a response to 0.4 g of hair or less. Normal responses are within the range of 1–2 g. All the staff involved in performing the behavioral tests and drug administration were blinded.

### Histologic analysis and OA scoring

Mice were euthanized at 3 weeks after MIA administration, and the knee joints were fixed immediately in 4% paraformaldehyde, decalcified in 10% EDTA for 14 days at 4 °C, and embedded in paraffin. Sagittal sections were cut at 4 μm thickness and then examined by toluidine blue and hematoxylin-eosin staining, and immunohistochemistry. Cartilage degeneration in the sections was assessed by three independent investigators using three histological scoring systems (cartilage degeneration score, aggrecan loss score, and Mankin score)^18,26,45^.

### Terminal dUTP nick-end labeling (TUNEL) assay

Decalcified paraffin-embedded sections of knee joints were processed for TUNEL assays using an in situ cell death detection kit (Roche Diagnostics Corp. 11684817910, Basel, Switzerland) following the manufacturer’s instructions. Apoptotic cells were determined by counting the percentage of TUNEL + cells in five 0.24 mm^2^ fields for each coded slide.

### Immunohistochemistry

Decalcified paraffin-embedded sections were individually incubated with primary antibodies to Beclin-1 (#3495, 1:200, Cell Signaling Technology Inc., Danvers, MA, USA) or LC3 (#12741, 1:200, Cell Signaling Technology Inc., Danvers, MA, USA), followed by incubation with the secondary antibodies and detected using a diaminobenzidine kit. All the sections were stained with hematoxylin as a nuclear stain. Beclin-1- or LC3-positive cells were counted using Image J software (NIH, Bethesda, MD, USA).

### Primary chondrocyte cultures

Normal articular cartilage was obtained from WT and KO mice euthanized under anesthesia. After knee joint surgery, articular cartilage pieces (0.5–1 mm^3^) were aseptically dissected from femoral head caps, and chondrocytes were obtained by incubation in 0.25% trypsin containing 0.02% EDTA for 15 min followed by digestion with collagenase II (2 mg/mL; Sigma-Aldrich, St. Louis, MO, USA) at 37 °C for 6 h. The cells were washed twice with phosphate-buffered saline (PBS) and cultured in DMEM/F-12 (Gibco, Carlsbad, CA, USA) supplemented with 10% (v/v) FBS and 100 units/mL of penicillin (Sigma-Aldrich, St. Louis, MO, USA) and streptomycin (Sigma-Aldrich, St. Louis, MO, USA) at 37 °C in a humidified atmosphere containing 5% CO_2_.

### Hoechst 33342 staining

Primary chondrocytes were seeded at 8 × 10^4^ cells per well and cultured for 24 h in 24-well plates. Chondrocytes were pretreated with nicotine in the absence or presence of the α7-nAChR‒selective antagonist, MLA, for 1 h and then treated with 5 μM MIA for 4 h. Cells were washed with PBS three times and fixed in 4% paraformaldehyde for 30 min at room temperature (22 ± 2 °C). After fixation, the cells were stained with Hoechst 33342 (10 μg/mL; AAT Bioquest, Sunnyvale, CA, USA) for 20 min and observed under a fluorescence microscope (Zeiss AX10; Carl Zeiss Microscopy GmbH, Jena, Germany). Cells displaying highly-condensed and brightly-stained nuclei were regarded as apoptotic cells. The apoptotic index was defined as the ratio of apoptotic cell number to total cell number.

### Flow cytometry

Apoptotic cells were quantified by flow cytometry using an Annexin V-FITC/PI Apoptosis Detection Kit (KeyGEN BioTECH Co. Ltd., Nanjing, China). Primary chondrocytes were digested with trypsin without EDTA and centrifuged at 1000 *rpm* for 5 min. Next, the cells were washed twice with PBS, centrifuged at 1000 *rpm* for 5 min, and gently resuspended in 500 μL binding buffer. After adding 5 μL annexin V-FITC and propidium iodide solutions, the cells were incubated in the dark for 10 min and analyzed by flow cytometry (Guava EasyCyte Plus, Merck-Millipore, Darmstadt, Germany) according to the manufacturer’s instructions.

### Measurement of chondrocyte mitochondrial membrane potential

Chondrocyte mitochondrial membrane potential (ΔΨm) was measured using the MitoProbeTMJC-1 assay kit for flow cytometry (Invitrogen Corp., Carlsbad, CA, USA). Primary cultured chondrocytes were treated as described above. Each sample was suspended in 1 mL warm PBS at ~1 × 10^6^ cells/mL and 10 μL of 200 μM JC-1 dye (2 μM final concentration) was added. The samples were incubated at 37 °C in a humidified atmosphere containing 5% CO_2_ for 20 min and then analyzed by flow cytometry (Guava EasyCyte Plus, Merck-Millipore, Darmstadt, Germany).

### Western blot analysis of mice chondrocytes and human specimens

Proteins were denatured in SDS sample buffer, separated by 10% SDS-PAGE, and transferred to nitrocellulose membranes using a wet transfer unit. The membranes were washed with Tris-buffered saline containing Tween-20 (TBST), blocked with 5% (w/v) milk in TBST at room temperature for 1 h, and incubated with primary antibodies overnight at 4 °C. Antibodies to Bcl-2 (#2876), Bax (#2772), cleaved caspase-3 (#9664), caspase-3 (#9665), the mammalian target of rapamycin (mTOR) (#2972), the phosphorylation of mTOR (p-mTOR) (#2971), Beclin-1 (#3495), p62 (#5114), and LC3 (#12741) (all diluted 1:1,000 and procured from Cell Signaling Technology Inc., Danvers, MA, USA); as well as antibodies to α7-nAChRs (#ANC007AN0402, 1:500, Alomone Labs, Jerusalem, Israel), SLURP-1 (#ab93840, 1:500, Abcam, Cambridge, MA, USA), Lynx1 (#ab125035, 1:500, Abcam, Cambridge, MA, USA), and β-actin (#BL005B, 1:10,000, Biosharp, China) were used. On the next day, the membranes were washed four times with TBST buffer and incubated with secondary antibody for 1 h, followed by four washes with TBST. Signals were detected using an enhanced chemiluminescence kit (Tanon 5200; Tanon Science and Technology Co. Ltd, Shanghai, China). The blots were scanned using a gel imaging system (Tanon Science and Technology Co. Ltd, Shanghai, China), and intensities of the protein bands were measured using Image J software (NIH, Bethesda, MD, USA).

### Statistical analysis

The sample size was chosen to ensure adequate power to detect a pre-specified effect size according to the previous reports^23^. All data are expressed as mean ± SEM. Statistical analyses were performed using one- GraphPad Prism software (San Diego, CA, USA). Human cartilage tissue protein data were normalized against β-actin, and differences calculated using Student’s unpaired *t* test. Mechanical withdrawal threshold was assessed via two-way repeated-measures analysis of variance (ANOVA) with time and treatment as factors followed by Tukey’s test. Other data were assessed with one-way or two-way ANOVA followed by Tukey’s test. *P* < 0.05 was considered to be statistically significant.
